# Case Report and Literature Review: Catastrophic Embolism Following Cosmetic Injection of Autologous Fat in the Face

**DOI:** 10.3389/fmed.2021.646657

**Published:** 2021-12-07

**Authors:** Chunyu Liu, Zhaolun Cai, Lingli Zhang, Muke Zhou, Li He

**Affiliations:** ^1^Department of Neurology, West China Hospital, Sichuan University, Chengdu, China; ^2^Department of Pharmacy, West China Second University Hospital, Sichuan University, Chengdu, China; ^3^Evidence-Based Pharmacy Center, West China Second University Hospital, Sichuan University, Chengdu, China; ^4^Key Laboratory of Birth Defects and Related Diseases of Women and Children, Sichuan University, Ministry of Education, Chengdu, China; ^5^West China School of Pharmacy, Sichuan University, Chengdu, China; ^6^Department of Gastrointestinal Surgery, West China Hospital, Sichuan University, Chengdu, China

**Keywords:** cosmetic fat injection in the face, autologous fat emboli, complications, visual loss, cerebral infarction

## Abstract

Injection of autologous fat on the face is a commonly performed procedure in plastic surgery. However, it can lead to rare but devastating complications due to fat embolism. In this study, we presented two cases of cerebral infarction and/or sudden vision loss after cosmetic injections of autologous fat on the face. Two women underwent injections into the temporal and frontal areas, respectively. In case 1, the patient underwent decompressive craniectomy as her condition deteriorated continuously and died. In case 2, the patient's vision had not improved at the 3-month follow-up visit. Imaging examinations showed occlusion of the right external carotid artery in case 1, and multiple retinal arterioles were segmentally occluded in case 2. We also screened relevant studies *via* a systematic search of PubMed (last updated on May 9, 2020) and performed a narrative review due to the significant heterogeneity between the studies. To prevent this catastrophic event, the autologous fat injection should be performed carefully. If embolization does occur, early diagnosis and timely treatment may help improve functional outcomes.

## Introduction

Injection of autologous fat on the face is a commonly performed procedure in plastic surgery (1). Although it is generally considered safe because the material obtained from the patient's fatty tissue is abundant and non-immunogenic (2, 3), there have been several reports on the literature of patients who suffered devastating complications such as sudden vision loss and cerebral infarction due to fat embolism following autologous fat injection on the face (2, 4–6).

In this study, we present two patients who suffered from sudden vision loss and cerebral infarction secondary to facial autologous fat injection. The principles outlined in the Declaration of Helsinki were followed. Occlusions of the external carotid artery and retinal arterioles were confirmed by angiography. We also reviewed the related literature to discuss the pathogenesis, potential risks, and follow-up treatment of these severe complications. Written informed consent for publication was obtained from the patients and their family representatives.

## Case Reports

### Case 1

An 18-year-old woman with no past medical history was referred to our hospital, complaining of sudden unconsciousness, left hemiplegia, and vomiting. Twenty-four hours before the onset of these symptoms, she had undergone an injection of autologous fat into the temporal area under local anesthesia, and the volume of the fat graft was unclear.

On arrival, her vital signs were normal. Neurological examination revealed lethargy, dysarthria, disorientation, and left-sided hemiplegia involving her lower face. Both pupils were fixed to the right side. Her left extremities exhibited hypotonia, weakened tendon reflex, and Medical Research Council (MRC) grade 2 status, while her right extremities were normal. Sensory examination showed hypoalgesia on the left. The Babinski reflex of the left side was positive.

**Figure 1:**
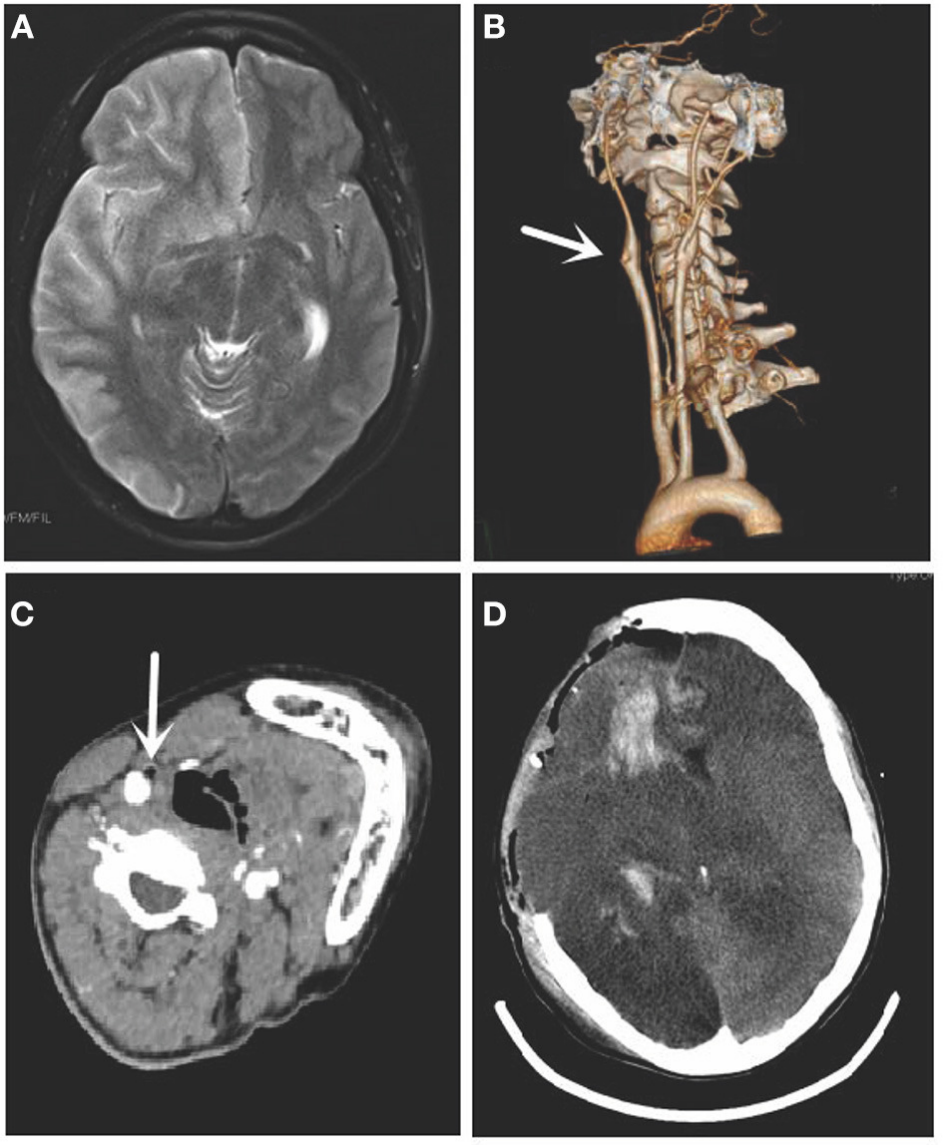
Magnetic resonance imaging (MRI), cervical computed tomography angiography (CTA), and computed tomography (CT) of case 1. **(A)** The cranial MRI showed acute infarction in the territory of the right internal carotid artery. **(B)** CTA showed occlusion of the right external carotid artery (arrow). **(C)** Cross-section indicated fat emboli within the lumen of the right external carotid artery (arrow). **(D)** Cranial CT showed the right swelling hemisphere and incomplete skull after decompressive craniectomy.

Magnetic resonance imaging (MRI) and computed tomography (CT) were performed immediately after admission. Cranial MRI showed acute infarction in the territory of the right internal carotid artery (Figure 1A). Cervical computed tomography angiography (CTA) showed occlusion of the right external carotid artery (Figure 1B). Cross-section indicated fat emboli within the lumen of the right external carotid artery (Figure 1C). Cranial MRI showed acute infarction in the territory of the right external carotid artery. No specific findings were seen in routine laboratory examinations.

The patient underwent decompressive craniectomy later, as her disorder of consciousness progressively worsened, and the pupils were asymmetrical. Cranial CT showed swelling of the right hemisphere and an incomplete skull after the operation (Figure 1D). However, her condition deteriorated continuously. She developed central respiratory failure and eventually died. Her family refused to grant permission for an autopsy. We obtained written informed consent from her family.

### Case 2

A 19-year-old woman with no past medical history was referred to our hospital complaining of sudden vision loss in the left eye and right hemiplegia. Four hours before the onset of these symptoms, she had undergone an injection of autologous fat into the frontal area under local anesthesia, and the volume of fat grafts was unclear. Immediately after the injection, she complained of severe periocular pain and blurred vision in her left eye. We obtained written informed consent from the patient.

On physical examination, the patient's level of consciousness was normal with stable vital signs. Her neurological examination revealed ideomotor apraxia of the right upper limb. Her right extremities appeared to have MRC grade 4 status, while her left extremities were normal. The left pupil was dilated and unresponsive to direct stimulation, and the right pupil was unresponsive to indirect stimulation. There was no light perception in the left eye.

**Figure 2:**
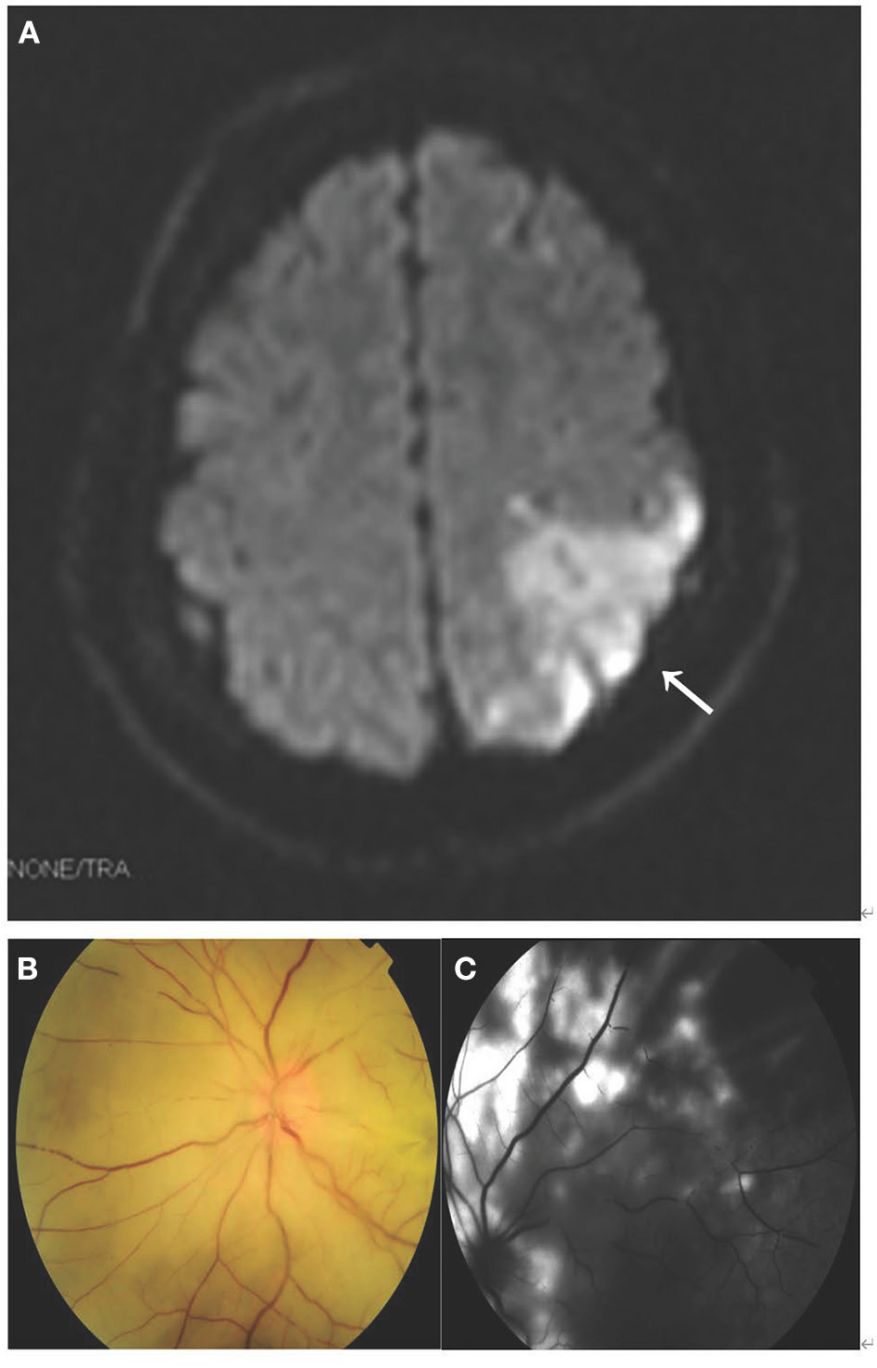
MRI, fundus photography, and fundus fluorescence angiography of case 2. **(A)** Diffusion-weighted MRI showed high signal intensity in the left parietal lobe (arrow). **(B)** Left funduscopic examination showed an edematous optic disc and widespread retinal whitening, with multiple retinal arterioles segmentally occluded by fat emboli. The posterior pole was pale yellow. **(C)** Left fluorescein angiography showed blockage of the retinal arterioles and lack of perfusion of the tissue bed in hypofluorescent areas.

Diffusion-weighted magnetic resonance imaging showed high signal intensity in the left parietal lobe (Figure 2A). Left funduscopic examination showed an edematous optic disc and widespread retinal whitening, with multiple retinal arterioles segmentally occluded by fat emboli. The posterior pole was pale yellow (Figure 2B). Left fluorescein angiography showed blockage of the retinal arterioles and a lack of perfusion of the tissue bed in hypofluorescent areas (Figure 2C). No specific findings were seen in electrocardiography, echocardiography, cranial magnetic resonance angiography, or cervical CTA. Laboratory examinations, such as screening for vasculitis and coagulopathies, were normal.

At the 3-month follow-up visit, the patient's vision had not improved.

## Discussion

Autologous fat injection is a common cosmetic procedure aimed at augmenting soft tissue defects by injecting the patient's fatty tissue (7). Although fat tissue is biologically reliable, there are many reported complications, such as edema, bruising, infection, lipogranuloma, and fat embolism (8). Fat embolism is relatively rare and extremely severe, which could result in vision loss and cerebral infarction (9).

Several authors have reported cases of vascular complications after facial autologous fat injections. However, a previous literature review did not well-document many details, such as risk factors, neurologic imaging or retinal imaging, and confused autologous fat with other types of filler. Moreover, none of the literature searches were comprehensive (10–13). Thus, we performed a thorough PubMed database search for relevant studies from its inception to May 9, 2020, and the search strategies were as follows: ((((facial filler injections) AND (autologous fat)) OR (autologous fat facial injection))) AND ((((((((Artery Embolism) OR (complication)) OR (Vision Loss)) OR (occlusion)) OR (cerebral infarction)) OR (Blindness)) OR (Embolism)) OR (Obstruction)) and reference lists. We also searched reference lists to identify additional potential studies (14).

A total of 26 articles were identified, and they were summarized in Supplementary Table 1 (1, 2, 4, 6, 10, 11, 15–34). Forty-one patients (35 women and 4 men; mean age 33.39 years old; range 18–66 years old) were included. It should be noted that complete demographic data were not available in three patients (21, 30). The most frequently injected sites were the glabella (10, 11, 15–17, 22, 31, 32), forehead (2, 10, 21, 24, 26, 30, 31), temple area (2, 6, 29, 31, 33, 34), and nasolabial groove (11, 18–21, 23). Two patients (4.9%) showed skin necrosis (25, 32). Associated initial ocular problems included ocular pain (39%) (1, 10, 11, 15, 17, 19, 20, 26, 31), ptosis (31.7%) (11, 16, 23, 24, 31), ophthalmoplegia (26.8%) (1, 11, 31), exotropia (14.6%) (1, 11), esotropia (2.4%) (11), and iris depigmentation (2.4%) (4). Nine cases (22.0%) involved retinal artery occlusion (11, 15, 18, 19, 21, 26, 29), and 19 cases (46.3%) involved ophthalmic artery occlusion (11, 16, 17, 20, 25, 28, 31, 32). Six (14.6%) cases involved brain infarction (17, 19, 20, 25, 32, 34), all of which were associated with middle artery infarction. Twenty-two (53.7%) cases involved therapies (1, 2, 6, 11, 15, 18, 22–25, 27–29, 32–34), mainly ocular massage (18, 25, 32), oxygen and carbon dioxide therapy (18, 25), anterior chamber paracentesis (11, 32), intravenous mannitol (2, 25), systemic steroids (22–24, 27), intraarterial thrombolysis (1, 11), and decompressive craniectomy (2, 6, 33, 34). However, none showed improvement in visual acuity.

The face is rich in blood vessels that have multiple branches and anastomoses with the external and internal carotid arteries. The internal carotid artery supplies blood to the forehead and nose. The ophthalmic artery is the first branch of the internal carotid artery. Its ocular group gives off the central retinal artery, posterior ciliary artery, and anterior ciliary artery. The central retinal artery travels through the optic nerve and supplies blood to the retina, while the posterior ciliary artery branches out to supply blood to the sclera and choroid.

Potential mechanisms of perioperative cerebral infarction in non-vascular surgeries include cerebral artery injury, decreased blood supply, increased coagulability after the operation, emboli from the heart or aorta, and air or fat embolisms (35). In our case report, stroke was most likely caused by fat embolism given the two patients' medical histories.

Many researchers have postulated that retrograde autologous fat is the main cause of arterial embolism (11, 13, 23). It has been reported that high-risk injection sites include the forehead, and glabellar, nasolabial fold, intranasal, periocular, and temporal areas (12, 30, 36). The excessive force and velocity of injection may result in an increase in local pressure. Pre-conditions, such as increased local pressure and well-vascularized tissue, are believed to be responsible for the intravasation of fat materials (37). Therefore, autologous fat was injected into the peripheral branches and moved distally into the main trunk of the ophthalmic artery in a retrograde manner (11). Once the injection ceased, the entering fat embolus was propelled by the blood flow in an anterograde manner and ultimately reached its distal branches, such as the central retinal artery (20). Furthermore, if the pressure generated was enough, the fat embolus could be pushed into the internal carotid artery in a retrograde flow, which could block the cerebral arteries and lead to cerebral infarction when released (38). Anastomoses between the external and internal carotid arteries may be active (11). This may explain why fat emboli could be detected in the external carotid artery.

Early recognition of fat embolism is essential for improving clinical outcomes and maximizing treatment efficacy. Central retinal artery occlusion may present with acute onset of ocular pain, vision loss, and visual field defects. Other signs include ptosis, exotropia, ophthalmoplegia, and pupillary defects (11, 12). Cerebral embolism may present with cranial nerve deficits and hemiparesis, which may occur immediately or after a few hours (39).

As the clinical outcomes are poor, it is important to minimize the risk of fat embolism. Fat injections should be performed gently and slowly with low pressure (2). Blunt needles may be the most appropriate instruments because sharp needles can perforate the wall of blood vessels and cannulate their lumen (33, 34). Using small syringes can help reduce stress on the plunger (38), and aspiration before the injection can help prevent intra-arterial injection (13). In addition, the plastic surgeons' technique, experience, and thorough knowledge of facial anatomy and common variants may also be crucial (39).

However, risks cannot be completely avoided. There is no safe and reliable treatment for reversing ophthalmic artery embolism to date (40). Once the patient develops symptoms associated with fat embolism, several actions should be taken immediately, and the patient should be transferred to a qualified hospital quickly. Treatment should be initiated within 90 min (12). In addition to the termination of autologous fat injection, treatment measures include ocular massage and breathing in a plastic bag (36). Ocular massage has been reported to be effective when performed frequently for over 3 h (36, 41). Thrombolytic agents have limited effects, as the fillers injected are fat, and mechanical thrombectomy may achieve vascular recanalization (1). Moreover, plastic surgeons should inform patients of the risk of these devasting complications (30).

## Conclusions

Our cases indicated that facial autologous fat injection can lead to devastating and even fatal complications in healthy adults. To prevent this catastrophic event, the autologous fat injection should be performed gently and slowly with low pressure, and blunt needles may be the most appropriate instruments. Meanwhile, thorough information should be given to patients before this procedure. If embolization does occur, early diagnosis and timely treatment may help improve functional outcomes.

## Data Availability Statement

The original contributions presented in the study are included in the article/Supplementary Material, further inquiries can be directed to the corresponding author/s.

## Ethics Statement

This study was approved by the Institutional Review Board of West China Hospital, Sichuan University. The patient gave written informed consent in accordance with the Declaration of Helsinki. Written informed consent was obtained from the patient for publication of the findings of this case report.

## Author Contributions

CL, MZ, and LH conceived the idea for this case report, carried out critical interpretations, and contributed to the final version of the article. CL and ZC collected the data, reviewed the literature, and wrote the article. CL prepared the figures and contributed to the revision of the manuscript. All authors read and approved the final version of the manuscript.

## Conflict of Interest

The authors declare that the research was conducted in the absence of any commercial or financial relationships that could be construed as a potential conflict of interest.

## Publisher's Note

All claims expressed in this article are solely those of the authors and do not necessarily represent those of their affiliated organizations, or those of the publisher, the editors and the reviewers. Any product that may be evaluated in this article, or claim that may be made by its manufacturer, is not guaranteed or endorsed by the publisher.

## References

[1] Park SJ Woo SJ Park KH Hwang JM Hwang GJ Jung C. Partial recovery after intraarterial pharmacomechanical thrombolysis in ophthalmic artery occlusion following nasal autologous fat injection. J Vasc Interv Radiol. (2011) 22:251–4. 10.1016/j.jvir.2010.10.023 21185202

[2] Wang DW Yin YM Yao YM. Internal and external carotid artery embolism following facial injection of autologous fat. Aesthet Surg J. (2014) 34:NP83–7. 10.1177/1090820X14539973 24936097

[3] Coleman SR. Structural fat grafting: more than a permanent filler. Plast Reconstr Surg. (2006). 118(3 Suppl):108S–20S. 10.1097/01.prs.0000234610.81672.e7 16936550

[4] Kim J Kim SK Kim MK. Segmental ischaemic infarction of the iris after autologous fat injection into the lower eyelid tissue: a case report. BMC Ophthalmol. (2017) 17:205. 10.1186/s12886-017-0599-8 29157214 PMC5697341

[5] Roshandel D Soheilian M Pakravan M Aghayan S Peyman GA. Middle cerebral artery, ophthalmic artery, and multibranch retinal vessel occlusion after cosmetic autologous fat transfer to forehead. Ophthalmic Surg Lasers Imaging Retina. (2015) 46:593–6. 10.3928/23258160-20150521-15 26057767

[6] Shen X Li Q Zhang H. Massive cerebral infarction following facial fat injection. Aesthetic Plast Surg. (2016) 40:801–5. 10.1007/s00266-016-0681-2 27439536

[7] Gir P Brown SA Oni G Kashefi N Mojallal A Rohrich RJ. Fat grafting: evidence-based review on autologous fat harvesting, processing, reinjection, and storage. Plast Reconstr Surg. (2012) 130:249–58. 10.1097/PRS.0b013e318254b4d3 22743888

[8] Sa H-S Woo KI Suh Y-L Kim Y-D. Periorbital lipogranuloma: a previously unknown complication of autologous fat injections for facial augmentation. Brit J Ophthalmol. (2011) 95:1259–63. 10.1136/bjo.2010.180547 21041459

[9] Çetinkaya A Devoto MH. Periocular fat grafting: indications and techniques. Curr Opin Ophthalmol. (2013) 24:494–9. 10.1097/ICU.0b013e3283634841 23925063

[10] Hong JH Ahn SJ Woo SJ Jung C Chang JY Chung JH. Central retinal artery occlusion with concomitant ipsilateral cerebral infarction after cosmetic facial injections. J Neurol Sci. (2014) 346:310–4. 10.1016/j.jns.2014.08.030 25201714

[11] Park SW Woo SJ Park KH Huh JW Jung C Kwon OK. Iatrogenic retinal artery occlusion caused by cosmetic facial filler injections. Am J Ophthalmol. (2012). 154:653–62.e1. 10.1016/j.ajo.2012.04.019 22835509

[12] Beleznay K Carruthers JD Humphrey S Jones D. Avoiding and treating blindness from fillers: a review of the world literature. Dermatol Surg. (2015) 41:1097–117. 10.1097/DSS.0000000000000486 26356847

[13] Lazzeri D Agostini T Figus M Nardi M Pantaloni M Lazzeri S. Blindness following cosmetic injections of the face. Plast Reconstr Surg. (2012) 129:995–1012. 10.1097/PRS.0b013e3182442363 22456369

[14] Cai Z Liu C Chang C Shen C Yin Y Yin X. Comparative safety and tolerability of approved PARP inhibitors in cancer: a systematic review and network meta-analysis. Pharmacol Res. 2021:105808. 10.1016/j.phrs.2021.105808 34389457

[15] Teimouria B. Blindness following fat injections. Plast Reconstr Surg. (1988) 82:361. 10.1097/00006534-198808000-00036 3399569

[16] Dreizen NG Framm L. Sudden unilateral visual loss after autologous fat injection into the glabellar area. Am J Ophthalmol. (1989) 107:85–7. 10.1016/0002-9394(89)90823-4 2912125

[17] Egido JA Arroyo R Marcos A Jiménez-Alfaro I. Middle cerebral artery embolism and unilateral visual loss after autologous fat injection into the glabellar area. Stroke. (1993) 24:615–6. 10.1161/01.STR.24.4.615 8465374

[18] Lee DH Yang HN Kim JC Shyn KH. Sudden unilateral visual loss and brain infarction after autologous fat injection into nasolabial groove. Br J Ophthalmol. (1996) 80:1026. 10.1136/bjo.80.11.1026 8976738 PMC505688

[19] Feinendegen DL Baumgartner RW Vuadens P Schroth G Mattle HP Regli F. Autologous fat injection for soft tissue augmentation in the face: a safe procedure? Aesthetic Plast Surg. (1998) 22:163–7. 10.1007/s002669900185 9618180

[20] Danesh-Meyer HV Savino PJ Sergott RC. Case reports and small case series: ocular and cerebral ischemia following facial injection of autologous fat. Arch Ophthalmol. (2001) 119:777–8. 11346413

[21] Coleman SR. Avoidance of arterial occlusion from injection of soft tissue fillers. Aesthet Surg J. (2002) 22:555–7. 10.1067/maj.2002.129625 19332014

[22] Yoon SS Chang DI Chung KC. Acute fatal stroke immediately following autologous fat injection into the face. Neurology. (2003) 61:1151–2. 10.1212/WNL.61.8.1151 14581689

[23] Park SH Sun HJ Choi KS. Sudden unilateral visual loss after autologous fat injection into the nasolabial fold. Clin Ophthalmol. (2008) 2:679. 10.2147/OPTH.S2782 19668775 PMC2694002

[24] Lee YJ Kim HJ Choi KD Choi HY. MRI restricted diffusion in optic nerve infarction after autologous fat transplantation. J Neuroophthalmol. (2010) 30:216–8. 10.1097/WNO.0b013e3181c5d147 20300009

[25] Lee CM Hong IH Park SP. Ophthalmic artery obstruction and cerebral infarction following periocular injection of autologous fat. Kor J Ophthalmol. (2011) 25:358–61. 10.3341/kjo.2011.25.5.358 21976947 PMC3178774

[26] Park YH Kim KS. Blindness after fat injections. N Engl J Med. (2011). 365:2220. 10.1056/NEJMicm1100804 22150040

[27] Lee KM Kim EJ Jahng GH Chang DI. Magnetic resonance findings in two episodes of repeated cerebral fat embolisms in a patient with autologous fat injection into the face. J Korean Neurosurg Soc. (2012) 51:312–5. 10.3340/jkns.2012.51.5.312 22792432 PMC3393870

[28] Xing L Almeida DR Belliveau MJ Hollands H Devenyi RG Berger A. Ophthalmic artery occlusion secondary to fat emboli after cosmetic nasal injection of autologous fat. Retina. (2012) 32:2175–6. 10.1097/IAE.0b013e31826a6897 23064430

[29] Lu L Xu X Wang Z Ye F Fan X. Retinal and choroidal vascular occlusion after fat injection into the temple area. Circulation. (2013) 128:1797–8. 10.1161/CIRCULATIONAHA.112.000397 24126325

[30] Carle MV Roe R Novack R Boyer DS. Cosmetic facial fillers and severe vision loss. JAMA Ophthalmol. (2014) 132:637–9. 10.1001/jamaophthalmol.2014.498 24604287

[31] Chen Y Wang W Li J Yu Y Li L Lu N. Fundus artery occlusion caused by cosmetic facial injections. Chin Med J. (2014) 127:1434–7. 24762584

[32] Hong DK Seo YJ Lee JH Im M. Sudden visual loss and multiple cerebral infarction after autologous fat injection into the glabella. Dermatol Surg. (2014) 40:485–7. 10.1111/dsu.12426 24447202

[33] Wang X Wu M Zhou X Liu H Zhang Y Wang H. Autologous fat used for facial filling can lead to massive cerebral infarction through middle cerebral artery or facial intracranial branches. J Craniofac Surg. (2018) 29:1341–3. 10.1097/SCS.0000000000004625 29863569

[34] Liu S Chen X Su Y Qiu L Chen X Yan D. Association of autologous fat injection in facial artery with ophthalmological complications: an experimental animal study. JAMA Facial Plast Surg. (2018) 20:445–51. 10.1001/jamafacial.2017.1975 29978203

[35] Tettenborn B Caplan LR Sloan MA Estol CJ Pessin MS DeWitt LD. Postoperative brainstem and cerebellar infarcts. Neurology. (1993). 43:471. 10.1212/WNL.43.3_Part_1.471 8450986

[36] Townshend A. Blindness after facial injection. J Clin Aesthet Dermatol. (2016) 9:E5. 28210400 PMC5300728

[37] Akhtar S. Fat embolism. Anesthesiol Clin. (2009) 27:533–50. 10.1016/j.anclin.2009.07.018 19825491

[38] Carruthers JD Fagien S Rohrich RJ Weinkle S Carruthers A. Blindness caused by cosmetic filler injection: a review of cause and therapy. Plast Reconstr Surg. (2014) 134:1197–201. 10.1097/PRS.0000000000000754 25415089

[39] Prado G Rodriguez-Feliz J. Ocular pain and impending blindness during facial cosmetic injections: is your office prepared? Aesthetic Plast Surg. (2017) 41:199–203. 10.1007/s00266-016-0728-4 28032150

[40] Parkinson T. Zyderm Collagen Implant Safety Notice. Collagen Corporation, (Palo Alto, California) (1983).

[41] Szantyr A Orski M Marchewka I Szuta M Orska M Zapała J. Ocular complications following autologous fat injections into facial area: case report of a recovery from visual loss after ophthalmic artery occlusion and a review of the literature. Aesthetic Plast Surg. (2017) 41:580–4. 10.1007/s00266-017-0805-3 28233134 PMC5440494

