# Supplementary material for: Case Report and Literature Review: Catastrophic Embolism Following Cosmetic Injection of Autologous Fat in the Face
Source: Front Med (Lausanne). 2021 Dec 7;8:646657. doi: 10.3389/fmed.2021.646657 (PMC8688397; doi:10.3389/fmed.2021.646657)
Supplement: Supplementary file 1 [file Table_1.docx]

| Reference | Age (yr) | Sex | Risk factors | Injection site | Symptoms | Time to visit | Affected Eye | | | | | Diagnosis | Treatment | Outcome  (Variable Time for Follow-up) |
| --- | --- | --- | --- | --- | --- | --- | --- | --- | --- | --- | --- | --- | --- | --- |
|  |  |  |  |  |  |  | **Side** | **Initial BCVA** | **Ophthalmic examinations** | **Fundoscopic examination** | **Fundus fluorescein angiography** |  |  |  |
| Teimourian 1988 | 45 | F | None | Glabella | Ocular pain and vision loss in the right eye | 2 days later | Right | NR | NR | NR | NR | RAO | Medication and support care | The vision had not improved. |
| Dreizen 1989 | 44 | F | NR | Glabella | Vision loss in the right eye and headache | 4 days later | Right | NLP | Blepharoptotic, proptosis  Pupil: nonreactive to direct light and a relative afferent pupillary defect | A pale optic disc and several retinal arterioles segmentally occluded | No filling of the retinal arterioles and minimal choroidal filling | OAO | NR | 2.5 months: The vision had not improved. The proptosis and conjunctival injection had completely resolved. |
| Egido 1993 | 47 | F | None | Glabella | Periocular pain and vision loss in the right eye and left hemiplegia | 2 hours later | Right | NLP | Restricted abduction, conjunctival injection  Pupil: nonreactive to direct light | A pale optic disc and several retinal arterioles segmentally occluded | NR | OAO and MCAO | NR | 3 weeks: The vision had not improved. The patient was able to raise the left leg against minimal resistance, while the left arm remained plegia. There was no anosognosia, and the left spatial neglect had improved. |
| Lee 1996 | 42 | F | NR | Nasolabial groove | Unconsciousness, headache and dyspnea | 2 hours later | Left | NR | Pupil: dilated and nonreactive to direct light | A cherry red spot on the macula, retinal ischemia and multiple fat emboli in the retinal arterioles | Incomplete filling of the narrowed left ophthalmic artery | CRAO and infarction of the left caudate head, thalamus and subcortical white matter of the left cerebral hemisphere | Ocular massage, oxygen and carbon dioxide therapy | 1 week: The patient was alert but there was no light perception in the left eye.  3 months: The vision had not improved. |
| Feinendegen 1998 | 45 | M | Bilateral neck and face rhytidectomy, liposuction of the cheeks, elevated triglyceride and reduced high-density lipoprotein (HDL) levels | Nasolabial grooves, lower lip and chin | Aphasia and right hemiparesis | 7 hours later | Right | NR | NR | Multiple fat emboli in the retinal and choroidal arterioles | NR | Occlusion of the right retinal and choroidal arterioles and the left middle cerebral artery | NR | 10 months: Fluent aphasia |
|  | 47 | F | Excision of a Baker’s cyst, removal of femoral varices, cosmetic correction of the inframammary folds, liposuction of the thighs, and a patent foramen ovale | Periorbital areas | Unconsciousness, ocular pain in the left eye and headache | NR | Left | NR | Pupil: dilated and weakly reactive to direct light | An edematous optic disc and retinal ischemia | NR | RAO and OAO, and infarctions in the left cerebral hemisphere at the border zones between the anterior and the middle as well as between the middle and the posterior cerebral arteries | NR | A few weeks: The patient was able to walk. The global aphasia had improved. The vision had not improved. |
| Danesh-Meyer 2001 | 43 | M | NR | The left side of the nose, nasolabial grooves, and upper and lower lips | Disorientation, aphasia, ocular pain and vision loss in the left eye, headache and right hemiparesis | NR | Left | NLP | Pupil: amaurotic | A pale optic disc, widespread retinal whitening and multiple fat emboli in the retinal arterioles | NR | OAO and MCAO | NR | 5 days: The vision had not improved. Neurologic deficits had improved to normal. There was patchy necrosis on the site of the initial injection. |
| Coleman 2002 | NR | NR | None | Cheek | Unilateral blindness | NR | NR | NR | NR | NR | NR | CRAO | NR | 5 days: There was a 3×2-cm vascular lesion on the ipsilateral side of the nose. |
|  | NR | NR | None | Each oral commissure, each lateral canthal area, each nasojugal trough， a transverse scar and wrinkle in the forehead | Unilateral blindness and headache | NR | NR | NR | NR | NR | NR | CRAO | NR | 4 days: There was a superficial skin lesion of the forehead. |
| Yoon 2003 | 39 | F | None | Glabella | Mental change, aphasia and right hemiplegia | Immediately | Left | NR | The eye was midline fixed.  Pupil: dilated and nonreactive to direct light | NR | NR | 0cclusion of the left internal carotid artery and infarction in the left cerebral hemisphere | Artificial ventilation and steroid therapy | 4 days: Died |
| Park 2008 | 27 | F | None | The right nasolabial groove | Vision loss in the right eye | NR | Right | Hand motion | Ptosis  Pupil: dilated and fixed | Multiple whitish patchy lesions with macular edema | Patchy choroidal filling | NR | Steroid therapy | 16 days: The vision had not improved.  6 months: The vision had not improved. |
| Lee 2010 | 24 | F | None | Forehead | Vision loss in the left eye, right parethesias and decreased sensation on the forehead and scalp | 3 days later | Left | NLP | Ptosis, restricted extraocular motility in all directions  Pupil: nonreactive to direct light and an afferent pupillary defect | An edematous optic disc and widespread retinal whitening | NR | MCAO and occlusion of the left optic nerve | Steroid therapy | 5 months: The vision had not improved. Ocular motility was normal except for mild adduction. |
| Lee 2011 | 44 | F | NR | Periocular area | Vision loss in the left eye and dysarthria | 2 hours later | Left | NLP | Pupil: dilated and a relative afferent pupillary defect | A cherry red spot on the macula and multiple fat emboli in the retinal arterioles | A delay of retinal arterial filling and prolonged choroidal filling | OAO and MCAO | Ocular massage, intravenous mannitol, and oxygen and carbon dioxide therapy | 30 minutes: The patient was able to speak normally.  24 hours and 2 months: The vision had not improved. |
| Park 2011 | 39 | F | None | The left side of the nose | Ocular pain and vision loss in the left eye | 1 hour later | Left | NLP | Ophthalmoplegia, large exotropia  Pupil: nonreactive to light | Multiple fat emboli and retinal ischemia | No filling of the retinal arterioles and minimal choroidal filling | OAO | Intraarterial thrombolysis | 4 days: The vision had not improved. The abduction of the left eye showed partially recovered.  3 months and 17 months: The vision had not improved. Ocular motility was normal except for mild exotropia. |
| Park 2011 | 32 | M | NR | Forehead | Periocular pain and vision loss in the left eye | 1 week later | Left | NLP | Pupil: dilated and nonreactive to direct light | An edematous optic disc, widespread retinal whitening and multiple fat emboli in the retinal arterioles | Multiple retinal hemorrhages and no filling of the tissue bed in hypofluorescent areas | RAO | NR | 2 months: The vision had not improved. |
| Lee 2012 | 26 | F | None | Face | Vision loss in the right eye and left hemiplegia | 13 hours later | Right | NR | The patient had a gaze palsy.  Pupil: dilated and nonreactive to direct light | NR | NR | Infarction in the right frontoparietal lobe, temporal lobe, frontal lobe, parietal lobe, caudate head, basal ganglia, genu of corpus callosum and subcortical white matters | Steroid therapy | NR |
| Park 2012 | 66 | F | NR | Left glabella | Ocular pain | Immediately | Left | NLP | Ptosis, ophthalmoplegia | NR | NR | OAO | Intra-arterial thrombolysis | 5 days: The vision had not improved. |
|  | 40 | F | NR | Left nasolabial groove | Ocular pain | Immediately | Left | NLP | Ophthalmoplegia, large exotropia | NR | NR | OAO | Intra-arterial thrombolysis | 511 days: The vision had not improved. |
|  | 18 | F | NR | Right nasolabial groove | Ocular pain | Immediately | Right | NLP | Ptosis, ophthalmoplegia, esotropia | NR | NR | OAO | Intra-arterial thrombolysis | 430 days: The vision had not improved. |
|  | 24 | F | NR | Left glabella | Ocular pain | 1 week later | Left | NLP | Ptosis, ophthalmoplegia, exotropia, relative afferent pupillary defect | NR | NR | OAO and MCAO | None | 63 days: The vision had not improved. |
|  | 37 | F | NR | Right glabella | Ocular pain | Immediately | Right | NLP | Ophthalmoplegia, exotropia, relative afferent pupillary defect | NR | NR | OAO | Anterior chamber paracentesis | 3 days: The vision had not improved. |
|  | 19 | F | NR | Left glabella | Ocular pain | 2 hours later | Left | NLP | Exotropia | NR | NR | OAO | Anterior chamber paracentesis | 40 days: The vision had not improved. |
|  | 26 | F | NR | Left glabella | NR | 2 days later | Left | Light perception | NR | NR | NR | CRAO, bilateral ACAO and left MCAO | Anterior chamber paracentesis | 16 days: The vision had not improved. |
| Xing 2012 | 23 | F | NR | The left side of the nose | Vision loss in the left eye | NR | Left | NLP | Pupil: an afferent pupillary defect | Widespread retinal whitening, a cherry red spot on the macula, and multiple fat emboli in the retinal arterioles | NR | OAO | Lower intraocular pressure | NA |
| Lu 2013 | 22 | M | NR | Right temple area | Vision loss in the right eye | 20 days later | Right | NLP | Pupil: an afferent pupillary defect | An atrophic optic disc, multiple lesions on the macula and narrowed retinal arterioles | A delay in arteriovenous transit time, no filling of the choroidal circulation and late leakage in the posterior pole | Chorioretinal infarction | Multivitamin therapy | Over 3 months: The vision had not improved. |
| Carle 2014 | Early 60s | F | None | The high part of the forehead | Vision loss in the right eye | On the same day | Right | NLP | NR | Widespread retinal whitening with lipid-filled arterioles | Incomplete filling of the retinal arterioles and patchy choroidal filling | NR | NR | NR |
| Chen 2014 | 24 | F | NR | Temple area | Ocular pain, ptosis, ophthalmoplegia and headache | NR | Right | NLP | NR | NR | NR | OAO | NR | 90 days: The vision had not improved. |
|  | 47 | F | NR | Forehead | Ptosis and ophthalmoplegia | NR | Left | NLP | NR | NR | NR | OAO | NR | 30 days: The vision had not improved. |
|  | 24 | F | NR | Glabella and frontal area | Vision loss in the left eye | NR | Left | NLP | NR | NR | NR | OAO | NR | 30 days: The vision had not improved. |
|  | 36 | F | NR | Forehead and temple area | Ocular pain, ptosis and ophthalmoplegia | NR | Right | NLP | NR | NR | NR | OAO | NR | 36 days: The vision had not improved. |
|  | 33 | F | NR | Periorbital areas, cheek, nose and lip | Ptosis and ophthalmoplegia | NR | Left | NLP | NR | NR | NR | OAO | NR | 25 days: The vision had not improved. |
|  | 27 | F | NR | Forehead and temple area | Ptosis, ophthalmoplegia, dizzy and vomiting | NR | Right | NLP | NR | NR | NR | OAO | NR | 11 days: The vision had not improved. |
|  | 27 | F | NR | Temple area | Ocular pain and ptosis | NR | Right | NLP | NR | NR | NR | OAO | NR | 55 days: The vision had not improved. |
| Hong 2014 | 27 | F | NR | Glabella, forehead and cheeks | Short-term memory disturbance, naming difficulty, periocular pain and vision loss in the left eye | NR | Left | NLP | Pupil: dilated and nonreactive to light | Widespread retinal whitening with narrow arterioles | A delay of retinal arterial filling | Left OAO and infarction in the left frontal lobe | NR | 1 year: The vision had not improved. Neurologic deficits had improved mildly. |
| Hong 2014 | 31 | F | NR | Glabella | Vision loss in the right eye and weakness in the left arm | 1 day later | Right | NR | NR | A pale optic disc, widespread retinal whitening and multiple fat emboli in the retinal arterioles | NR | OAO and MCAO | Ocular massage, anterior chamber paracentesis and volume expansion | 1 week: The vision had not improved. Neurologic deficits had improved to normal.  5 months: The vision had not improved. |
| Wang 2014 | 22 | F | None | Temple and frontal areas | NR | 4 hours later | Left | NR | The eye was midline fixed.  Pupil: dilated and nonreactive to direct light | NR | NR | OAO, MCAO, ACAO and occlusion of the left internal and external carotid arteries | Decompressive craniectomy and intravenous mannitol | 1 day: The patient was alert and developed aphasia, vision loss in the left eye and right hemiplegia.  1 month: The patient was able to raise the right leg against minimal resistance, while the right arm remained paretic.  2 months: The vision had not improved. The aphasia had improved. |
| Shen 2016 | 30 | F | None | Bilateral temple areas and chin | Unconsciousness, left hemiplegia, several episodes of urinary incontinence and vomiting | 8 hours later | Right | NR | Pupil: nonreactive to light | NR | NR | NR | Lower intracranial pressure, volume expansion and decompressive craniectomy | NR |
| Kim 2017 | 28 | F | None | Face | Segmental iris depigmentation in the right eye | NR | Right | Normal | NR | Normal | Normal | NR | NR | 2 months: The vision had not improved. |
| Wang 2018 | 22 | F | None | Bilateral temple areas | Unconsciousness and left hemiplegia | NR | NR | NR | NR | NR | NR | MCAO | Decompressive craniectomy | Discharge: left hemiplegia, ageusia and vision loss in both eyes  2 years: The patient could walk and move the left arm without assistance.  33 months: There was squint and slight ptosis in the right eye. The left temporal lobe was apparently depressed. |
|  | 30 | F | Elevated high myocardial troponin I, myohemoglobin and CK-MB | Bilateral temple areas | Left hemiplegia | NR | NR | NR | NR | NR | NR | Infarction in the right cerebral hemisphere | Decompressive craniectomy | Left hemiplegia |
| Liu 2019 | 42 | F | Slightly elevated serum albumin, triglyceride concentrations, and white blood cell count | Temple area | Unconsciousness, global aphasia and right hemiplegia | Immediately | NA | NA | Pupil: dilated | No fat emboli in the retinal arterioles | NR | MCAO | Decompressive craniectomy | 5 days: The patient was alert.  2 years: Complete expressive aphasia and right hemiplegia |

Note:

NLP = no light perception; RAO = retinal artery occlusion; OAO = ophthalmic artery occlusion; CRAO = central retinal artery occlusion; MCAO = middle cerebral artery occlusion; ACAO = anterior cerebral artery occlusion
